# Magnesium Oxychloride Cement Composites with Silica Filler and Coal Fly Ash Admixture

**DOI:** 10.3390/ma13112537

**Published:** 2020-06-03

**Authors:** Adam Pivák, Milena Pavlíková, Martina Záleská, Michal Lojka, Ondřej Jankovský, Zbyšek Pavlík

**Affiliations:** 1 Department of Materials Engineering and Chemistry, Faculty of Civil Engineering, Czech Technical University in Prague, Thákurova 7, 166 29 Prague 6, Czech Republic; adam.pivak@fsv.cvut.cz (A.P.); milena.pavlikova@fsv.cvut.cz (M.P.); martina.zaleska@fsv.cvut.cz (M.Z.); pavlikz@fsv.cvut.cz (Z.P.); 2 Department of Inorganic Chemistry, Faculty of Chemical Technology, University of Chemistry and Technology, Technická 5, 166 28 Prague 6, Czech Republic; michal.lojka@vscht.cz

**Keywords:** magnesium oxychloride cement, coal fly ash, sand substitution, structural properties, mechanical resistance, hygric and thermal parameters, thermal stability

## Abstract

Worldwide, Portland cement-based materials are the most commonly used construction materials. As the Portland cement industry negatively affects the environment due to the excessive emission of carbon dioxide and depletion of natural resources, new alternative materials are being searched. Therefore, the goal of the paper was to design and develop eco-friendly, low-cost, and sustainable magnesium oxychloride cement (MOC)-based building material with a low carbon footprint, which is characterized by reduced porosity, high mechanical resistance, and durability in terms of water damage. To make new material eco-efficient and functional, silica sand which was used in the composition of the control composite mixture was partially replaced with coal fly ash (FA), a byproduct of coal combustion. The chemical and mineralogical composition, morphology, and particle morphology of FA were characterized. For silica sand, FA, and MgO, specific density, loose bulk density, and particle size distribution were measured. Additionally, Blaine specific surface was for FA and MgO powder assessed. The workability of fresh mixtures was characterized by spread diameter. For the hardened MOC composites, basic structural, mechanical, hygric, and thermal properties were measured. Moreover, the phase composition of precipitated MOC phases and their thermal stability were investigated for MOC-FA pastes. The use of FA led to the great decrease in porosity and pore size compared to the control material with silica sand as only filler which was in agreement with the workability of fresh composite mixtures. The compressive strength increased with the replacement of silica sand with FA. On the contrary, the flexural strength slightly decreased with silica sand substitution ratio. It clearly proved the assumption of the filler function of FA, whereas its assumed reactivity with MOC cement components was not proven. The water transport and storage were significantly reduced by the use of FA in composites, which greatly improved their resistance against moisture damage. The heat transport and storage parameters were only slightly affected by FA incorporation in composite mixtures.

## 1. Introduction

From the beginning of the 20th century, Portland cement (PC) has become the most extensively used construction material worldwide due to the concrete and various types of cement-based composites production. At present, PC is the most-produced and consumed substance on Earth, with a world average annual production of about 4.6 billion metric tons [1,2]. The building industry is, therefore, related to significant environmental impact. Approximately 8% of worldwide anthropogenic carbon dioxide (CO_2_) emissions are linked with cement and concrete industry, while cement is responsible for about 95% of the emissions released during concrete production [3]. Recently, increasing concerns over global CO_2_ emissions lead to proposals for many alternative binders with a low carbon footprint, which would represent a kind of counterbalance to concrete- and cement-based composites [4,5]. This group of materials also includes reactive magnesia cement (MgO-based cement), which is a versatile material that can be used as a binder or together with other materials in various applications such as magnesium oxychloride cement (MOC), magnesium phosphate cement (MPC), magnesium silicate hydrate (M-S-H) cement, etc. [6]. MgO-based cements are considered low-carbon materials, thanks to their CO_2_ sequestration ability and low calcination temperature (the typical calcination temperatures to produce light-burn MgO are 900–1050 °C) [6,7]. It was reported for MOC-based materials that they are able to offset the CO_2_ emissions during the carbonation, and the final net emitted CO_2_ linked with the whole life cycle of MOC is, therefore, 40–50% lower than that associated with Portland cement manufacturing [8]. Moreover, as a result of the carbonation of MOC-based composites due to the CO_2_ sequestration, improvement of mechanical parameters thanks to the formation of a denser microstructure and a micro-hardness was stated [8,9,10,11,12,13].

Magnesium oxychloride cement was first introduced by Sorel in 1867 and belongs to nonhydraulic materials [10]. MOC is formed during the chemical reaction between caustic calcined magnesite powder (MgO) and a solution of magnesium dichloride (MgCl_2_). The obtained product is a ternary composite salt MgO-MgCl_2_-H_2_O with the main crystalline phases, present at ambient temperature, of 3Mg(OH)_2_∙MgCl_2_∙8H_2_O (Phase 3) and 5Mg(OH)_2_∙MgCl_2_∙8H_2_O (Phase 5) [14,15,16,17].

Superior properties of MOC-based composites include: rapid setting and hardening, high early mechanical parameters, high abrasion, and fire resistance, and also the ability to integrate both organic and inorganic aggregate in a large amount [18,19,20]. Currently, to the main applications of MOC belong flooring, panels for fire protection, thermal and sound isolation, in addition to decorative purposes, grinding wheels, and thanks to the beige/ivory color, ornamental applications and stucco [21]. 

Due to the nature of an air-dried binder, MOC exhibits poor water resistance. This limits their wider use in civil engineering [8,22]. To improve this property, many studies have been conducted using phosphates [23], tartaric and phosphoric acids [24], or acrylic emulsion [25]. However, the usage of these chemicals increases the production costs of final MOC materials. In order to optimize the pore structure, improve water resistance, and also decrease the cost of MOC-based composites, fly ash (FA) as waste from coal combustion is the subject of extensive research at present [26,27,28]. Fly ash, which is often used in a concrete production all over the world, is easily accessible in a required amount, and there exist well-developed methods for its characterization [29]. In the studies aimed at its incorporation in MOC-based composites, FA is usually reused as the replacement/addition by weight of MgO. However, FA can also serve as an “inert” microfiller of MOC composites, replacing partly silica filler in order to both enhance the properties of final material and save resources of natural silica sand.

In this respect, MOC-based composites with partial replacement of silica sand with FA in the ratio of 5%, 10%, and 15% by volume were prepared and investigated in both fresh and hardened states to evaluate their workability, basic structural, mechanical, hygric, and thermal parameters. The replacement ratio of silica sand with FA was chosen because of the high accumulation capacity of MOC and the assumed filler effect of fine FA particles in MOC/silica sand mixture.

The main aim of this study was to develop eco-friendly, low-cost MOC-based material with a low carbon footprint, which is characterized by reduced porosity, high mechanical properties, and improved behavior in terms of water-induced damage. The thermal behavior and properties of developed composites were also studied as they present unique information for future research.

## 2. Materials and Methods 

For the preparation of MOC-based composites, caustic MgO powder (Styromagnesit Steirische Magnesitindustrie Ltd., Oberdorf, Austria), MgCl_2_∙6H_2_O of p.a. purity (Lach-Ner, Ltd., Neratovice, Czech Republic), silica sand, and coal fly ash were used. Standardized silica sand was produced by Filtrační písky Ltd. (Chlum u Doks, Czech Republic). It was composed of three sand fractions of specific particle size range; PG1 (0.063–0.5 mm), PG2 (0.5–1.0 mm), and PG3 (1.0–2.0 mm). The particular sand fractions were mixed in a weight ratio of 1:1:1. Fly ash coming from coal power plant Opatovice Inc. (Pardubice, Czech Republic) was used as partial replacement of silica sand. The replacement ratio was 5%, 10%, and 15% by volume.

The chemical composition of quartz sand, FA, and commercially delivered MgO were tested on XRF (X-ray fluorescence) principle using an ED-XRF spectrometer ARL QUANT′X (Thermo Fisher Scientific, MA, USA). The software UniQuant 5 (Thermo Scientific, Milan, Italy) was used to collect and analyze the measured data. The typical dry sample mass was 7–10 g. The data provided by XRF analysis is in the form of oxides shown in Table 1. Based on the measured chemical composition and in accordance with the ASTM C 618 [30], the analyzed FA was classified into Class F. It can be also designated as low calcium FA.

**Figure 1:**
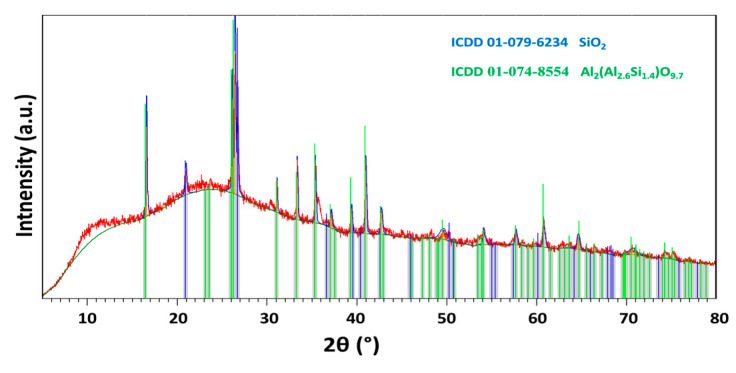
X-ray diffractogram of FA.

X-ray powder diffraction (XRD) of FA was carried out using a Bruker D2 Phaser (Bruker AXS GmbH, Karlsruhe, Germany), a powder diffractometer with Bragg–Brentano geometry, applying CuKα radiation (λ = 0.15418 nm, U = 30 kV, I = 10 mA) and rotation (five rounds per minute). The step size was set to 0.02025° (2θ), and the overall data was acquired in the angular range of 5°–80°. The obtained diffractogram is shown in Figure 1. According to XRD, the sample was highly amorphous, from crystalline phases it contained mainly quartz (ICDD 01-079-6234) and aluminum–silicon oxide (ICDD 01-074-8554).

**Figure 2:**
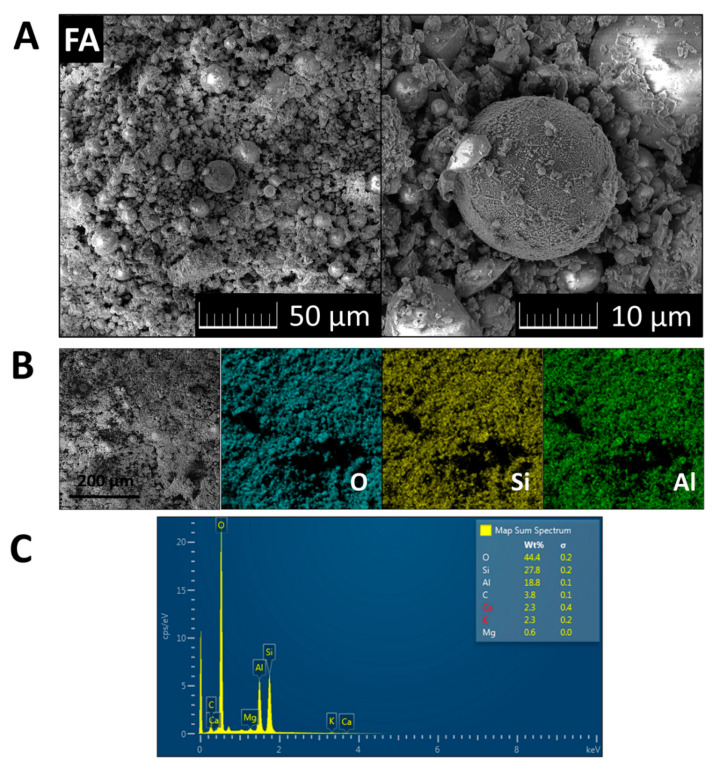
(**A**) SEM micrographs of FA; (**B**) EDS elemental maps of major elements; (**C**) and EDS spectrum.

The morphology of FA was investigated using scanning electron microscopy (SEM) with a FEG electron source (Tescan Lyra dual-beam microscope, Tescan Brno, s.r.o., Brno, Czech Republic). Elemental composition and mapping were performed using an energy dispersive spectroscopy (EDS) analyzer (X-MaxN) with a 20 mm^2^ SDD detector (Oxford instruments, High Wycombe, UK) and AZtecEnergy software. To conduct the measurements, the samples were placed on a carbon conductive tape. SEM and SEM-EDS measurements were carried out using a 10 kV electron beam. SEM and EDS data are presented in Figure 2. Typical morphology was obtained, ash contained cenospheres with dimensions between 3 and 20 μm. EDS elemental maps of major elements are also shown in Figure 2. According to EDS, the following elements were present in the sample: Al, Si, O, C, Ca, K, and Mg. These results confirmed the presence of phases detected by XRD, and were in agreement with data provided by XRF analysis.

For MgO, silica sand, and FA, specific density and loose bulk density were measured. The specific density was measured on a Pycnomatic ATC (Porotec, Hofheim, Germany) helium pycnometer. The loose bulk density was calculated based on the dry mass of the sample in the container of a known volume. For FA and MgO, Blaine specific surface was accessed in accordance with the standard EN 196-6 [31]. The basic physical parameters of tested raw materials are given in Table 2.

**Figure 3:**
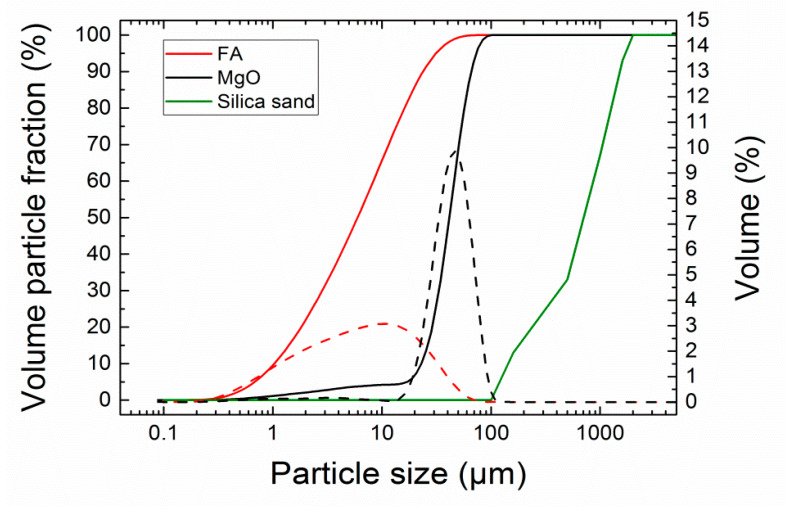
Particle size distribution of FA, MgO, and silica; dashed lines—incremental distribution curves, solid lines—cumulative curves sand.

The particle size distribution of FA and MgO powder was analyzed by a laser diffraction method; an Analysette 22 Micro-Tec plus (Fritsch GmbH, Industriestraße 8, Idar-Oberstein, Germany) device with two semiconductor lasers, green (*λ* = 532 nm, 7 mW) and IR (*λ* = 940 nm, 9 mW), was used. Both FA and MgO were measured 5 times (5 scans) and particle size distribution was created from the average values (see Figure 3). The grain size distribution of silica sand was assessed in the standard sieve test conducted in compliance with the EN 933-1 [32]. Fly ash was much finer compared to MgO; the particle size distribution parameters were following: d_10_ = 1.06 μm, d_50_ = 6.24 μm, d_90_ = 23.12 μm for FA, and d_10_ = 23.01 μm, d_50_ = 41.63 μm, d_90_ = 66.80 μm for MgO. The grain size of silica sand corresponded to the used fractions of the standardized sand.

The weight proportioning of the composites mixtures is given in Table 3, where acronym MOC-Ref stands for control composite with silica sand as only filler, MOC-FA5 stands for MOC composite with 5% substitution of silica sand with FA by volume, and MOC-FA10 and MOC-FA15 represent composites with 10% and 15% silica sand replacements, respectively. The mixing and preparation of composites were performed in compliance with the standard EN 14016-2 [33]. As the standard EN 14016-2 prescribes composition of MOC mortars based on pure MgO, the content of MgO in prepared composites was increased in order to enhance the reactivity of MOC binder. The water/binder ratio was 0.26 and similar for all mixtures.

The plastic molds were oiled with mineral oil to prevent leakage during filling and mortar sticking. Then they were filled in two layers. Each layer was compacted for 30 s by the use of vibration plate BS VIB 02 R (Beton System, Brno, Czech Republic). The casted samples were standard prisms having dimensions 40 mm × 40 mm × 160 mm. The hardened composite samples were removed from the molds (left freely in laboratory at T = (23 ± 2) °C, RH = (50% ± 5%) after 24 h, and left to cure for 27 d under the laboratory conditions at T = (23 ± 2) °C, RH = (50% ± 5%)). As MOC is an aerial binder, the samples were not covered in order to ensure the evaporation of batch water which was precisely dosed in accordance with the EN 14016-2 [33].

The workability of fresh composite mixtures was verified using a flow table test which was realized in compliance with the EN 1015-3 [34].

For hardened materials, phase composition, structural, mechanical, hygric, and thermal parameters were assessed. The thermal stability of developed materials was also tested.

As the presence of silica sand would overlap the XRD peaks of formed MgO phases, MOC pastes with FA as only filler were tested. The MOC-FA pastes were mixed, casted, and cured similarly as composite mixtures, i.e., they were tested after 28 d maturing at T = (23 ± 2) °C, RH = (50% ± 5%). X-ray powder diffraction of MOC pastes was carried out similarly as in the analysis of FA.

Among structural parameters of researched composites, bulk density, specific density, and porosity were measured. For each composite mixture, 5 samples were tested. The dry bulk density *ρ_b_* (kg∙m^−3^) was determined according to the standard EN 1015-10 [35] with the expanded combined uncertainty of 1.4%. For the measurement, the halves of casted prisms were used. The specific density *ρ_s_* (kg∙m^−3^) was measured by helium pycnometry (see above). The typical dry sample mass was about 40 g. The samples were dried at 60 °C in a vacuum dryer Vacucell (BMT, Brno, Czech Republic). The expanded combined uncertainty of the specific density test was 1.2%. Based on the bulk density and specific density data, the porosity *P* (%) was calculated [36]. The expanded combined uncertainty of the porosity assessment was 2.0%.

For examined materials, mechanical resistance was characterized by flexural strength, compressive strength, and Young’s modulus measurements. The tests of mechanical parameters were conducted on originally casted prisms. The flexural strength of composites *R_f_* (MPa) was determined using a standard three-point-bending test. The compressive strength *R_c_* (MPa) was measured on the far edge of the specimen fragments from flexural strength testing. The loading area was 40 mm × 40 mm. Both strength tests were carried out in compliance with the EN 1015-11 [37]. The dynamic modulus of elasticity (Young´s modulus) *E_d_* (GPa) was obtained in the ultrasound velocity test using instrument Pundit Lab+ (Proceq, Schwerzenbach, Switzerland) with a couple of 54 kHz transducers. The expanded combined uncertainty of this method was 2.3%. The relationship between *E_d_* (GPa), wave velocity *ν* (m∙s^−1^), and bulk density *ρ_b_* (kg∙m^−3^) is given in Equation (1)

(1) $$E_{d}=\rho_{b}\pi \nu^{2}$$

The microstructure of hardened composites was analyzed also by mercury intrusion porosimetry (MIP). The pore size distribution measurement was performed with Pascal series mercury porosimeters Pascal 140 and Pascal 440 (Thermo Fisher Scientific). The dry sample mass was approx. 1 g.

As the MOC-based materials are usually susceptible to moisture damage, the hygric parameters of composites were tested. The 24 h water absorption *W_a_* (%) was accessed in accordance with the EN 13755 [38]. The expanded combined uncertainty of the water absorption test was 1.2%. The water transport in studied composites was described by the water absorption coefficient and apparent moisture diffusivity. The water absorption coefficient *A_w_* (kg∙m^−2^∙s^−1/2^) was measured in the free water uptake experiment [39] in accordance with EN 10115-18 [40]. Based on the assessed *A_w_* values and the maximum of the imbibition curve of water, the apparent moisture diffusivity *κ* (m^2^∙s^−1^) was calculated. The details on the experimental setup and calculation procedure can be found in [41,42]. The expanded combined uncertainty of the water absorption test was 2.3%, and that of the apparent moisture diffusivity test was 3.5%. The sorption capacity of examined composites for water vapor was tested using a DVS Advantage II (Surface Measurement Systems, Allentown, PA USA), dynamic vapor sorption apparatus for sorption and desorption isotherms measurement. The measurement was realized in an automatic mode, based on continuous measurement of sample mass variation over time at specific relative humidity steps. Following relative humidity steps were set in the sorption/desorption test: 20%, 40%, 60%, 80%, and 90%.

For the measurement of thermophysical parameters of tested materials, thermal constant analyzer Hot Disk TPS 1500 (Hot Disk AB, Göteborg, Sweden) was applied. When performing the measurement, a Kapton-insulated sensor was fitted between two parallel pieces of the tested sample. In our case, the dry specimens having a square cross-section (40 mm × 40 mm) and a length of 80 mm were tested. As the moisture presence significantly affects thermal material properties, the samples were dried at 60 °C in a vacuum chamber until the difference in sample mass was for 2 consecutive measurements < 0.1%. The test was performed under the laboratory conditions at *T* = (23 ± 2) °C. Based on the hot disk measurements, the thermal conductivity *λ* (W∙m^−1^∙K^−1^), the thermal diffusivity *a* (m^2^∙s^−1^), and the volumetric heat capacity *c_v_* (J∙m^−3^∙K^−1^) were obtained.

The thermal behavior of investigated composites was analyzed by Simultaneous thermal analysis (STA). The DTA and TG curves were recorded simultaneously on a Linseis STA PT1600 (Linseis Messgeraete GmbH, Selb, Germany) apparatus at a heating rate of 5 °C∙min^−1^ in a dynamic air atmosphere (50 mL∙min^−1^).

## 3. Results and Discussion

**Figure 4:**
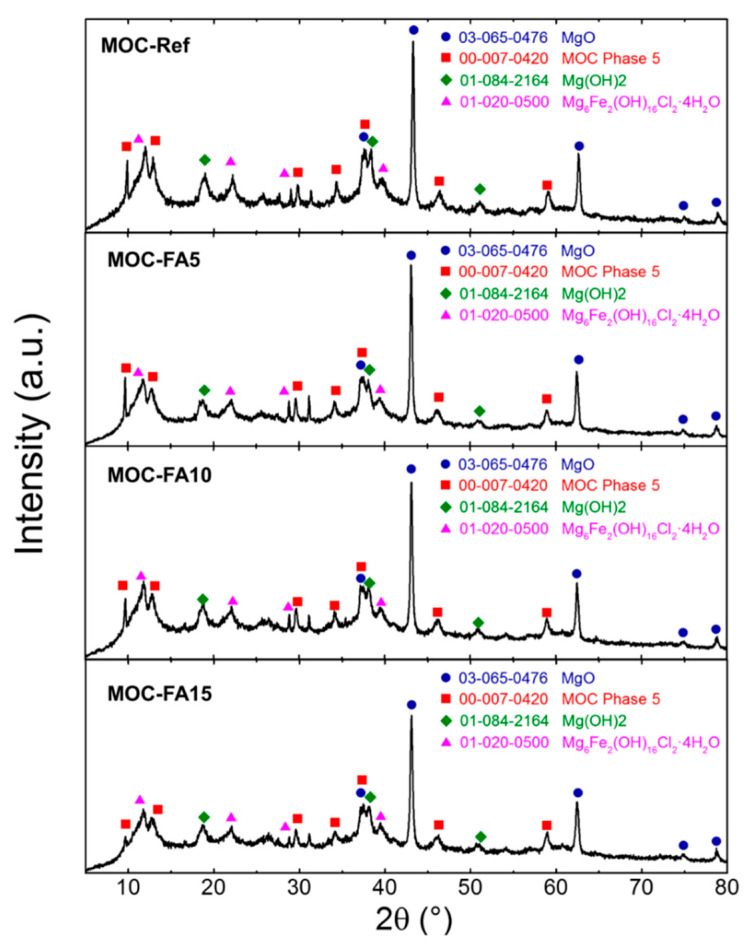
XRD. X-ray diffraction pattern for pastes MOC-Ref, MOC-FA5, MOC-FA10, and MOC-FA15.

The phase composition of all four paste samples was very similar as can be seen from Figure 4. The intensities of individual reflections decreased when increasing the amount of FA. This was caused due to the amorphous nature of FA. All samples contained magnesia (ICDD 03-065-0476), magnesium hydroxide (brucite, ICDD 01-084-2164), and MOC Phase 5 (Mg_3_(OH)_5_Cl 4H_2_O, ICDD 00-007-0420). Due to the presence of iron oxide in starting magnesia, phase iowaite was formed (Mg_6_Fe_2_(OH)_16_Cl_2_·4H_2_O, ICDD 00-020-0500).

The spread diameter of fresh mortar mixtures and the structural parameters of developed MOC-based composites are summarized in Table 4. For 5% replacement of silica sand with FA, the spread diameter slightly increased which pointed to the improvement in the rheology of the fresh mixture. The workability of composites with a higher dosage of FA was slightly worse than that observed for control mixture MOC-Ref. It was caused by two contradictory effects: (i) improvement of packing density in case of MOC-FA5 mixture; (ii) higher fineness and thus batch water imbibition of FA compared to those of silica sand in materials MOC-FA10 and MOC-FA15, respectively. The partial substitution of silica sand with coal FA led to a significant drop in porosity. In contrast to the bulk density that was only slightly changed by the use of FA, the specific density was greatly reduced with the dosage of FA in the composite mixture. The lowest porosity was obtained for material MOC-FA5. In this case, the decrease in porosity was 61.9% compared to reference composite MOC-Ref. It was the consequence of its improved consistency that enabled better workability and thus compaction during casting which resulted in a denser MOC structure. On the other hand, worsening in the workability of mixtures MOC-FA10 and MOC-FA15 brought slightly higher porosity than obtained for MOC-FA5. However, the differences in porosity values were very low.

The parameters characterizing the mechanical resistance of hardened composites are introduced in Table 5. All tested composites exhibited high mechanical strength and Young’s modulus which is typical for materials on MOC basis [26,43,44]. The high strength of MOC composite prepared is due to the high surface area (acicular structure) of MOC and its excellent bonding characteristics due to which it can migrate deep into the aggregate surface and forms interparticle and intersurface friction [45]. A high flexural/compressive strength ratio and high Young’s modulus are also typical technical properties of MOC-based materials [46]. The flexural/compressive strength ratio which was in our case 0.29–0.26 is superior property to ordinary Portland cement that usually has a flexural/compressive strength ratio in the range of 0.1–0.2 [47]. The admixing of FA led to an increase of the compressive strength of composite MOC-FA5 compared to the control material MOC-Ref. This can be attributed to the lower porosity of this material and changes in the pore volume of pores affecting strength which was the result of the filler effect of FA admixture. For higher silica sand substitutions, the compressive strength remained almost similar to that of MOC-Ref. The filler effect of FA was for a higher sand replacement ratio surpassed by the contribution of the lower compressive strength of FA itself compared to that of silica sand. This can be anticipated from loose bulk density and specific density values of FA and silica sand, respectively. Based on XRD analysis, no MOC/FA precipitated phases were identified, which means FA acted as filler only. This is the reason for slightly lower flexural strength and Young’s modulus in comparison with control material. However, taking into consideration the measurements’ uncertainties, the differences in *R_f_* values were negligible.

**Figure 5:**
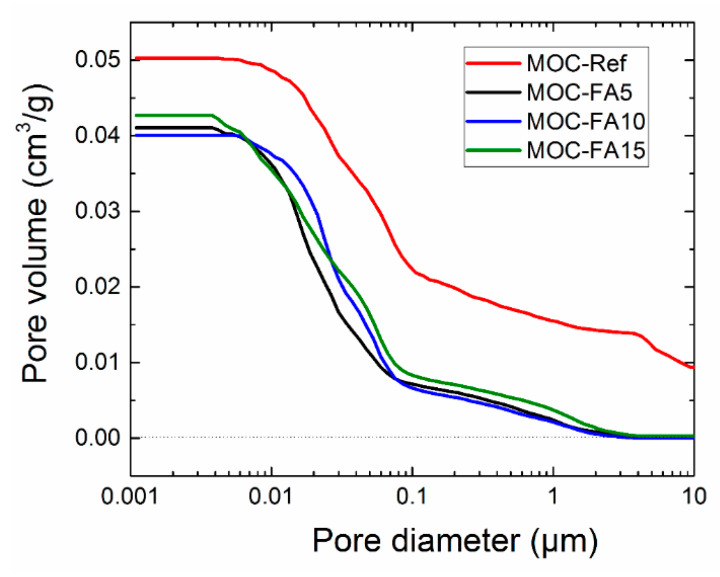
Cumulative pore volume curves.

The cumulative pore volume curves measured by MIP analysis of hardened composites are shown in Figure 5. In addition, distribution in pore volume is obvious from Table 6. The cumulative pore volume curves were in agreement with the porosity data as control material exhibited the highest pore volume over the whole investigated pore diameter range (0.001–100 μm). There is apparent that there is not yet a distinct terminology used for the various sizes of pores in construction composites. In this paper, we adopted the classification of pore sizes introduced for cement-based materials by Mindess et al. [48] and Mehta [49]. The highest volume of macropores affecting strength and permeability of final composites was identified for control material MOC-Ref. On the other hand, the incorporation of FA in composites structure greatly improved the packing of fresh composite mixtures and thus densified the microstructure of hardened materials. The pores in FA modified materials included micropores (“gel pores”) and small, medium, and large capillaries. The volume of macropores was negligible and their presence can be assigned to entrained air. In all MOC-FA materials, medium capillaries responsible for strength and permeability prevailed. This clearly corresponded with data on the mechanical and structural parameters presented above. In the reduction of porosity and pore size, the filler effect of fine FA particles was dominant. It was the result of the low particle size of FA and filling the gaps between the binder acicular crystals with FA cenospheres.

Susceptibility of MOC-based materials to water damage is generally considered the greatest disadvantage of these types of materials. However, only materials that enable water imbibition can be deteriorated. The liquid water transport in examined materials was characterized by their hygric parameters summarized in Table 7. The use of FA significantly reduced the rate of water transport and absorption, which is beneficial for their durability, and thus practical use. In comparison with the control composite, the decrease in the water absorption coefficient was 54.1% for MOC-SF5, 50.8% for MOC-FA10, and 45.9% for MOC-FA15. The relative drop in the 24 h water absorption *W_a_* was 35.8 for MOC-FA5, 32.7% for MOC-FA10, and 28.0% for MOC-FA15. Quantitatively, all assessed hygric properties were for all MOC-FA materials very low, which was the result of their reduced porosity and pore size.

**Figure 6:**
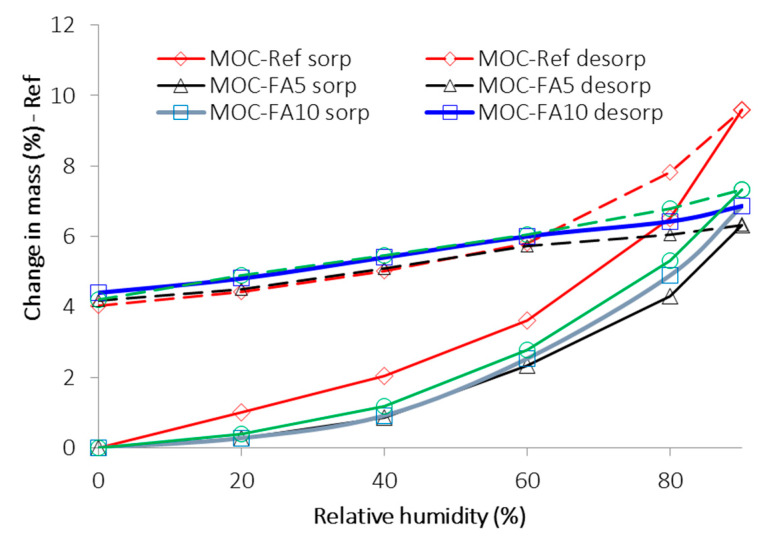
Sorption and desorption isotherms.

The plot of DVS isotherms is presented in Figure 6. Both sorption and desorption isotherms are introduced. Most of the reported sorption isotherms can be classified into one of the six isotherms classes originally formulated by Brunauer et al. [50,51]. According to Hansen [52], in porous construction materials, the adsorption isotherms are almost exclusively of type II [50,51], i.e., with an S-shape. However, it was not our case. All measured sorption isotherms were of type III, which is typical for very small interactions between adsorbent and the adsorbed medium. The maximum change in mass of the sample, i.e., the maximum mass of water vapor adsorbed by studied composites, ranged from ~6.4% for MOC-FA5 to ~9.6% for reference material MOC-Ref. The differences among particular composites were due to the variations in porosity, pore size distribution, shape of pores, and heterogeneity of their surface. It is quite apparent the use of FA decreased the water vapor adsorption capability of tested materials which is positive for their stability and thus durability. All examined materials exhibited high hysteresis in the desorption process. Surprisingly the lowest values of mass change in measured desorption curves were almost similar for all composites. The hysteresis phenomenon can be explained by the radius of capillary condensation, the ink-bottle or pore-blocking theory, and contact angle hysteresis [53,54].

**Figure 7:**
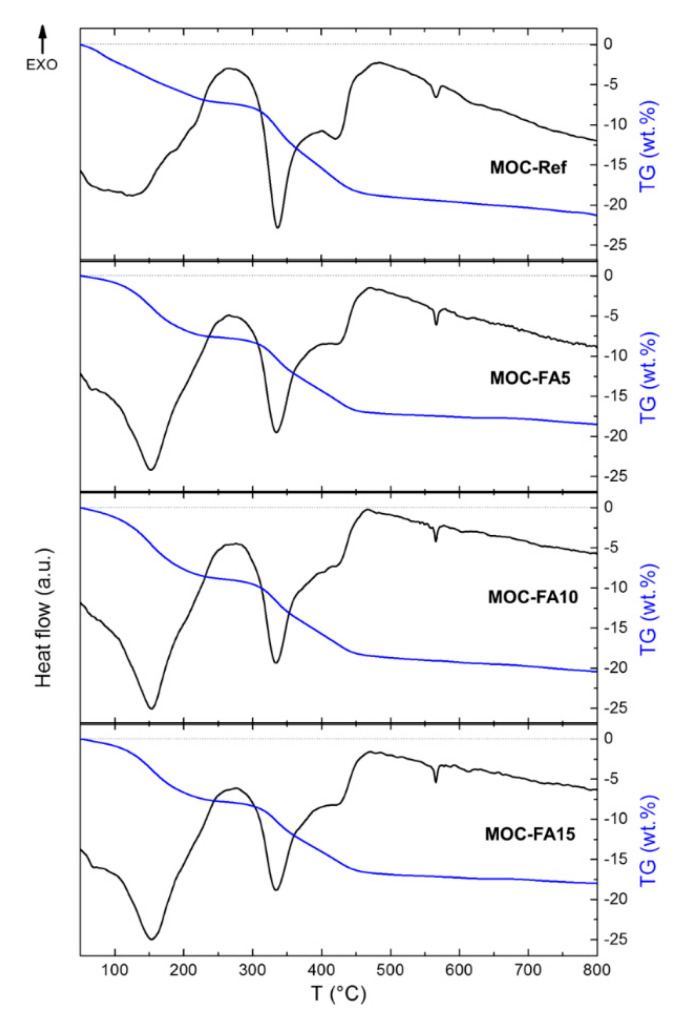
DTA and TG curves of MOC-Ref, MOC-FA5, MOC-FA10, and MOC-FA15.

The thermal behavior of prepared MOC-FA pastes and the reference sample MOC-Ref was measured by STA (Figure 7). In this case, MOC-FA pastes were tested in lieu of composites with silica sand aggregate as it affects the thermal decomposition of MOC precipitated phases in specimens of low mass. If part of specimen mass would contain silica sand, the resolution of STA analysis and identification of decomposition reactions would be more difficult and less precise. Very similar behavior was obtained for all three samples. The samples gradually decomposed to magnesia, while water and hydrochloric acid were released in a gaseous form during the heating [55,56,57]. These results were in good agreement with the literature, where the detailed mechanism of MOC decomposition was described [17]. According to the TG curve, the total weight decrease was in the range of 18–21%. The small endothermic effect starting at ~570 °C was due to the quartz inversion (quartz origin is from FA), where α-quartz undergoes a reversible structural transition to *β*-quartz.

The heat transport and storage parameters of tested materials measured on a transient hot disk method principle are given in Table 8. The presented values correspond to materials fully dried in a vacuum chamber at 60 °C. Two contradictory effects influenced the resulting thermal properties. In MOC-FA5, the decrease in porosity led to the acceleration of heat transport and greater heat storage compared to the reference material MOC-Ref. On the other hand, in materials MOC-FA10 and MOC-FA15, the porosity effect was compensated by the low thermal conductivity of used FA, which can be anticipated based on its low loose bulk density. In these materials, the contribution of silica sand substitution by FA to the overall thermal performance of examined composites prevailed over the impact of decreased porosity. From the quantitative point of view, the measured thermal conductivity and thermal diffusivity values were high. Unlike numerous studies on the testing of heat transport and storage in PC- and lime-based composites, information on thermal properties of MOC composites were rarely reported yet. Certain exception represents work published by Záleská et al. [22]. Here the authors analyzed MOC composite having a porosity of 13.4% and the compressive of 63.2 MPa. For this material, they reported a thermal conductivity of 2.09 W∙m^−1^∙K^−1^. Therefore, high thermal conductivity of low porosity MOC composites can be expected in practical applications.

## 4. Conclusions

Structural, mechanical, hygric, and thermal parameters of newly designed and developed MOC-based composites were tested and discussed. In order to support and ensure the sustainability of the construction industry, fly ash, a byproduct of coal combustion in power plants, was used as a partial substitute of natural silica sand in the composition of investigated composites. MOC was used as a representative of low carbon binders which is due to its low calcination temperature and high CO_2_ uptake capacity of precipitated MOC phases.

Based on the conducted tests and acquired data, the following conclusions were drawn:

- The main precipitated phases in hardened composites were magnesium hydroxide (brucite), MOC Phase 5 and iowaite;
- The use of FA led to a significant decrease in porosity and microstructure refinement compared to the control material with silica sand as only filler;
- For 5% replacement of silica sand with FA the spread diameter slightly increased; the workability of composites with a higher dosage of FA was slightly worse than that measured for control mixture MOC-Ref;
- The mechanical resistance of MOC-FA composites was high; the compressive strength was for 5% silica sand substitution even higher than that of the reference composite;
- The water transport and storage were greatly reduced by the use of FA in composite mixes;
- The water vapor sorption capacity of materials with FA decreased compared to reference material; contrary to that all tested composites yielded similar and high hysteresis in desorption;
- Heat transport and accumulation in designed composites were high as a result of porosity and the thermal behavior of incorporated FA.

Because the MOC-FA composites exhibited interesting functional parameters and performance, they may represent the basis of modern alternative building materials that could be designed in such a way to meet all the technical, financial, and environmental requirements of building practice and present society. The main benefits of the developed materials are low carbon footprint, reuse of industrial byproduct in the production process, and their specific properties which can find use in the manufacturing of eco-friendly and sustainable construction products. Moreover, the use of silica sand alternative might reduce the price of MOC-based materials and depletion of natural resources especially in areas, where silica aggregate is no longer available. In future research, a more detailed investigation of the durability of the developed materials is necessary with respect to their practical use in the building industry. In these studies, the freeze/thaw resistance and possible water damage will be of particular importance.

## Figures and Tables

**Table 1:** Chemical composition of caustic MgO, silica sand, and fly ash (FA; wt%).

Substance	MgO	Silica Sand	FA
SiO_2_	3.8	96.3	50.3
CaO	5.2	–	1.8
Al_2_O_3_	6.0	3.1	35.4
MgO	80.5	0.4	1.4
Fe_2_O_3_	3.8	–	6.8
K_2_O	–	–	1.7
SO_3_	0.4	–	0.4
Na_2_O	–	–	0.1
TiO_2_	–	0.1	1.5
P_2_O_5_	–	–	–
∑	99.7	99.9	99.4

**Table 2:** Physical parameters of MgO, silica sand, and fly ash.

Material	Loose Bulk Density (kg∙m^−3^)	Specific Density (kg∙m^−3^)	Blaine Specific Surface (m^2^∙kg^−1^)
MgO	841	3338	701
Silica sand	1660	2650	–
Fly ash	793	2061	407

**Table 3:** Composition of magnesium oxychloride cement (MOC) composites (g).

Material	MgO	MgCl_2_	Water	Silica Sand (PG1, PG2, PG3)	Fly Ash
MOC-Ref	1541.25	682.85	567.25	3 × 1125.00	–
MOC-FA5	1541.25	682.85	567.25	3 × 1068.75	95.54
MOC-FA10	1541.25	682.85	567.25	3 × 1012.50	191.07
MOC-FA15	1541.25	682.85	567.25	3 × 956.25	286.61

**Table 4:** Workability and basic structural parameters of developed composites.

Material	Spread Diameter (mm)	*ρ_b_* (kg∙m^−3^)	*ρ_s_* (kg∙m^−3^)	*P* (%)
MOC-Ref	165/165 ± 5	2124 ± 30	2430 ± 29	12.6 ± 0.3
MOC-FA5	170/170 ± 5	2170 ± 30	2280 ± 27	4.8 ± 0.1
MOC-FA10	160/160 ± 5	2137 ± 30	2257 ± 27	5.3 ± 0.1
MOC-FA15	160/160 ± 5	2124 ± 30	2250 ± 27	5.6 ± 0.1

**Table 5:** Mechanical parameters of developed composites.

Material	*R_f_* (MPa)	*R_c_* (MPa)	*E_d_* (GPa)
MOC-Ref	22.9	77.7	40.3
MOC-FA5	22.1	81.0	37.5
MOC-FA10	22.0	77.6	37.4
MOC-FA15	19.9	77.2	36.9

**Table 6:** Distribution of pore volume.

Pore Diameter Range (μm)	Specific Vol. (cm^3^/g)	Specific Vol. (%)	Relative Vol. (%)
MOC-Ref			
100–10	0.00936	18.59	18.59
10–1	0.01542	30.60	12.01
1–0.1	0.02186	43.40	12.80
0.1–0.01	0.04845	96.18	52.78
0.01–0.001	0.05027	99.79	3.61
MOC-FA5			
100–10	0.00007	0.16	0.16
10–1	0.00220	5.33	5.17
1–0.1	0.00706	17.13	11.80
0.1–0.01	0.03566	86.51	69.38
0.01–0.001	0.04106	99.61	13.10
MOC-FA10			
100–10	0.00005	0.12	0.12
10–1	0.00197	4.89	4.77
1–0.1	0.00649	16.14	11.25
0.1–0.01	0.03717	92.51	76.37
0.01–0.001	0.04008	99.78	7.27
MOC-FA15			
100–10	0.00027	0.63	0.63
10–1	0.00354	8.30	7.67
1–0.1	0.00817	19.13	10.83
0.1–0.01	0.03490	81.69	62.56
0.01–0.001	0.04268	99.91	18.22

**Table 7:** Water transport and storage parameters of developed composites.

Material	*A_w_* (kg∙m^−2^∙s^−1/2^)	*κ* (m^2^∙s^−1^)	W_a_ (%)
MOC-Ref	0.061	4.20 × 10^−9^	3.21
MOC-FA5	0.028	6.66 × 10^−10^	2.06
MOC-FA10	0.030	8.64 × 10^−10^	2.16
MOC-FA15	0.033	1.14 × 10^−9^	2.31

**Table 8:** Thermal parameters of developed composites.

Material	*λ* (W∙m^−1^∙K^−1^)	*a* × 10^−6^ (m^2^∙s^−1^)	*c_v_* × 10^6^ (J∙m^−3^∙K^−1^)
MOC-Ref	3.030	1.318	2.302
MOC-FA5	3.173	1.484	2.139
MOC-FA10	2.996	1.315	2.273
MOC-FA15	2.920	1.448	1.996
